# Comparing the efficacy and pregnancy outcome of intrauterine balloon and intrauterine contraceptive device in the prevention of adhesion reformation after hysteroscopic adhesiolysis in infertile women: a prospective, randomized, controlled trial study

**DOI:** 10.1186/s12958-024-01222-w

**Published:** 2024-04-23

**Authors:** HaiXia Ding, Honghong Zhang, Rui Qiao, Ningxia Sun, Yixuan Ji, Wenjuan Pang, Wen Li, Qing Zhang

**Affiliations:** 1 Center for Reproductive Medicine & Fertility Preservation Program, The International Peace Maternity and Child Health Hospital, School of Medicine, Shanghai Jiao Tong University, Shanghai, 200030 China; 2 Shanghai Key Laboratory of Embryo Original Diseases, Shanghai, 200030 China; 3 Reproductive Medicine Center, Second Military Medical University, Changzheng Hospital, Shanghai, People’s Republic of China; 4 Reproductive Medicine Center, The First Affiliated Hospital, Zhejiang University School of Medicine, Hangzhou Zhejiang, People’s Republic of China

**Keywords:** Intrauterine adhesion, Intrauterine balloon, Intrauterine contraceptive device, Hysteroscopy, Pregnancy outcome

## Abstract

**Study objective:**

To evaluate the efficacy and pregnancy outcomes of intrauterine balloon and intrauterine contraceptive devices in the prevention of adhesion reformation following hysteroscopic adhesiolysis in infertile women with moderate to severe intrauterine adhesion.

**Design:**

A prospective, randomized, controlled trial study.

**Setting:**

A tertiary university hospital.

**Patients:**

A total of 130 patients with moderate (American Fertility Society [AFS] score of 5–8) and severe (AFS score of 9–12) intrauterine adhesions were recruited.

**Interventions:**

86 patients were evenly allocated to group treated with an IUD for 1 month and group treated with an IUD for 2 months. 44 patients were allocated to group treated with a Foley catheter balloon.(IUD: Yuangong IUD).

**Measurements and main results:**

The primary outcome measures were the AFS score, endometrial thickness, and pregnancy outcome. After hysteroscopy, the AFS score was significantly decreased(*P*<0.05), whereas endometrial thickness was significantly increased across the three groups(*P*<0.001). Notably, the decline in the AFS score in the balloon group was greater than that in the IUD-1-month group and IUD-2-month group(*P*<0.01), with no significant difference between the IUD groups(*P* = 0.298). Lastly, In addition, the extent of the increase in endometrial thickness(*P* = 0.502) and the pregnancy outcomes(*P* = 0.803) in the three groups were not significantly different.

**Conclusion:**

Inserting a balloon or placing an IUD for one or two months can effectively lower the risk of adhesion recurrence and restore the shape of the uterine cavity. While the therapeutic effect of the balloon was superior to that of the IUD, no significant differences were observed in the one-month and two-month IUD groups.

**Trial registration:**

This research was registered in the Chinese Clinical Trial Registry (http://www.chictr.org.cn/enIndex.aspx); Clinical trial registry identification number: ChiCTR-IOR-17,011,943 (http://www.chictr.org.cn/showprojen.aspx?proj=17979). Date of trial registration: July 11, 2017.

## Introduction

Intrauterine adhesion (IUA) is caused by trauma, infection, and any other factors that lead to injury of the endometrial basal layer, dysfunctional repair of the endometrium, and fibrosis of the endometrium, eventually resulting in partial or complete obstruction of the uterine cavity, and/or the cervical canal [1]. Its most common cause is dilation and curettage (D&C), especially in a gravid uterus. Indeed, approximately 93% of IUA cases are caused by curettage after miscarriage [2]. IUAs are also associated with infection involving the uterine cavity, intrauterine interventions, genital tuberculosis, and congenital anomalies of the uterus [3].

IUA was first reported by Fritsch in 1894, and Asherman detailed descriptions of its etiology, pathological features, and clinical manifestations in 1948. Therefore, it is also referred to as Asherman syndrome (AS) [1] and is characterized by reduced menstrual volumes, amenorrhea, periodic abdominal pain, secondary infertility, recurrent miscarriage, placental implants, and other clinical symptoms [4, 5]. At present, the incidence of female infertility in the general population is 9–18% [6], with IUAs being a prevalent cause of secondary infertility [7]. IUA has become one of the most persistent disorders affecting the reproductive prognosis and quality of life of reproductive-age women.

The objective of treatment is to restore the normal volume and shape of the endometrial cavity, treat and manage related symptoms, prevent readhesion, promote endometrial regeneration, and restore fertility in women of childbearing age [8]. The main challenge of hysteroscopic adhesiolysis is its high rate of reformation of adhesions, especially in patients with severe adhesions, wherein the recurrence rate may attain 62.5% [4]. A number of strategies have been proposed to mitigate the recurrence of adhesions postoperatively. Currently, the placement of an IUD in the uterine cavity has been the traditional approach to maintaining the patency of the uterine cavity and is frequently used to prevent adhesion formation after adhesiolysis. It is typically removed after a few months. IUDs can accelerate physiological endometrial regeneration by separating the surface of the wound after adhesiolysis, with numerous studies reporting favorable outcomes [9, 10]. However, there is currently no universal standard for the placement of IUDs. In addition, the placement of a Foley catheter balloon in the uterine cavity for 5 to 7 days following surgical lysis of IUAs has also been described to prevent readhesion [11]. Foley catheter balloons are not only able to separate the surface of the wound but also minimize the risk of infection by draining the internal hemorrhage and inflammatory exudate within the uterine cavity.

In this study, we share our experience with the use of three different methods to prevent the recurrence of IUAs following the treatment of severe cases, namely a Foley catheter balloon, an IUD for one month, and an IUD for two months.

## Materials and methods

### Subjects

This study was approved by the institutional review board of Changzheng Hospital, Shanghai, China (Approval number: 2020shenglun-038-01). All participants provided written informed consent prior to participation in the series. The study was carried out at the reproductive medicine center of the hospital from January 2021 to May 2021. A total of 130 women diagnosed with severe IUAs based on the AFS IUA scoring system (AFS 1988 version) (The American Fertility Society, 1988), as listed in Table 1, were included in this study [12]. All patients were infertile women who experienced embryo transfer failures. Prior to the surgical intervention, all patients underwent preoperative evaluations, including a detailed history of the menstrual pattern, previous intrauterine surgeries, reproductive history, and transvaginal ultrasonography. The exclusion criteria were women aged over 40 years who suffered from other uterine diseases, such as uterine fibroids, uterine adenomyosis, and uterine malformation, and those with significant medical disorders, including thrombophilia and cardiovascular and respiratory diseases.

Randomization of the groups was performed in a 1:1:1 ratio using a computer-generated randomization sequence; the randomization results were sealed in opaque envelopes securely stored in a closed study box. The randomization sequence was concealed until the intervention was assigned by physicians-investigators in this study.

**Table 1:** The American Fertility Society (AFS) classification of intrauterine adhesions, 1988

Affected area	<1/3	1/3 to 2/3	>2/3
	1	2	4
Adhesions	Filmy	Filmy and dense	Dense
	0	2	4
Menstrual pattern	Normal	Hypomenorrhoea	Amenorrhoea
	0	2	4
Stage of adhesion			
Stage I (mild)	1–4		
Stage II (moderate)	5–8		
Stage III (severe)	9–12		

Source: The American Fertility Society classification of adnexal adhesions, distal tubal occlusion, tubal occlusion secondary to tubal ligation, tubal pregnancies Mullerian anomalies, and intrauterine adhesions. Fertility Steril;49:944 − 55

### Sample size calculation

Assuming an adhesion reformation rate of 40% in the IUD-1-month group and IUD-2-months group and 15% in the balloon group, with a type 1 error (α) of 0.05 and a type 2 error (β) of **0.20**, each group of the randomized, controlled trial would require 43 patients. Considering a drop-out rate of 10%, the total number of patients required to be recruited was estimated at 130 .(IUD: Yuangong IUD).

## Procedure

### Hysteroscopy

The surgical procedure was carried out by an experienced hysteroscopic surgeon using a hysteroscope (Storz) perfused with saline solution under a pressure ranging from 120 mmHg to 140 mmHg. The procedure was performed under general anesthesia. After the degree and severity of uterine adhesion were assessed, they were separated with the use of hysteroscopic scissors until normal uterine anatomy was restored. Following the intervention, the patients were randomly allocated to receive one of the following three treatments: (A) placing an IUD into the uterine cavity for one month; (B) placing an IUD into the uterine cavity for two months; and (C) placing a balloon into the uterine cavity for 5 days. (After verification, we have made a commitment that the economic expenses for the treatment method are within the acceptable range for the subjects) In other words, a total of 130 patients were randomly divided into three groups. According to the chronological order of diagnosis and treatment, the 130 patients were randomly assigned to either the experimental group or the control group according to the random allocation table.

### Postoperative treatment

#### Antibiotic therapy

All subjects were intravenously administered a combination of sulbenicillin 4 g and levornidazole for 1 day to lower the risk of infection.

#### Hormone therapy

Hormone therapy was initiated after the operation, consisting of estradiol valerate at a dose of 4 mg per day for 21 days and dydrogesterone at a dose of 10 mg per day for the last 10 days of estradiol valerate therapy. Following the withdrawal bleed, the hormone therapy was repeated for an additional cycle.

#### Second-look hysteroscopy

For women who underwent intrauterine balloon insertion, the device was removed after 5 days, and a second-look hysteroscopy was carried out during the early proliferative phase, 1 month after the initial operation. For women who had an IUD fitted after the initial hysteroscopic procedure, the device was removed during the second-look hysteroscopy, approximately 1–2 months after the initial operation. After the initial inspection, the degree and severity of recurrent intrauterine adhesions were documented.

#### Pregnancy outcomes

Pregnancy outcomes of embryo transfer after the second-look hysteroscopy were recorded.

### Statistical analysis

Statistical analyses were performed using SPSS version 20.0. The reduction in the AFS score and the improvement in endometrial thickness across the three groups were compared using one-way ANOVA. The pregnancy rate in the three groups was compared using the X^2^ test. A P value of < 0.05 was considered statistically significant.

## Results

**Fig. 1:**
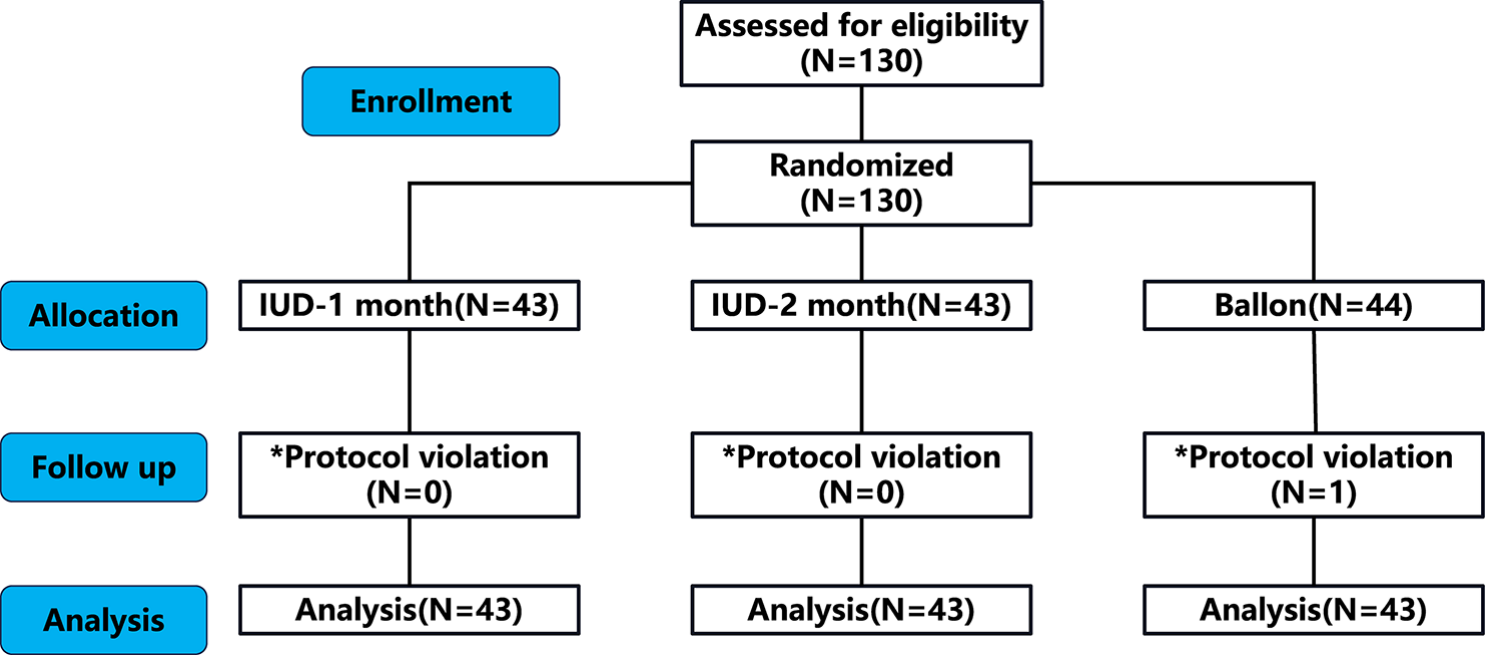
CONSORT flow diagram. *Protocol violation was defined as not undergoing a second-look hysteroscopy within the pre-defined time.

A total of 130 patients were randomized, but 1 subject was excluded owing to protocol violation (1 in the balloon group) (Fig. 1). The general conditions and clinical characteristics of each group are detailed in Table 2. Notably, the preoperative baseline parameters and clinical outcomes were comparable among the three groups.

**Table 2:** General Conditions and Clinical Characteristics. There was no significant difference in the per-operative baseline parameters and clinical outcomes between the three groups

General Conditions and Clinical Characteristics					
	Total	IUD-1month	IUD-2month	Balloon	P value
	129	43	43	43	
Age		33.42 ± 4.69	32.45 ± 4.65	24.37 ± 3.08	0.382
BMI		23.6 ± 2.71	22.92 ± 3.48	22.17 ± 2.23	0.076
Number of D&C		1.34 ± 1.18	13.06 ± 1.08	1.13 ± 1.27	0.166
**Symptom**					
Oligomenorrhea	73	31 (72%)	30 (70%)	32 (73.3%)	0.767
Normal menses	36	12 (28%)	13 (30%)	11 (26.7%)	
AFS scores		7.48 ± 1.49	7.66 ± 1.69	7.79 ± 1.4	0.073
Endometrial thickness		7.05 ± 1.67	7.40 ± 1.73	7.06 ± 1.50	0.500

AFS = American Fertility Society; D and C = dilation and curettage; IUA = intrauterine adhesion. Values are expressed as mean ± standard deviation and number (%) unless otherwise indicated. One-way ANOVA test; p-value of < 0.05 was considered statistically significant

### AFS score

**Fig. 2:**
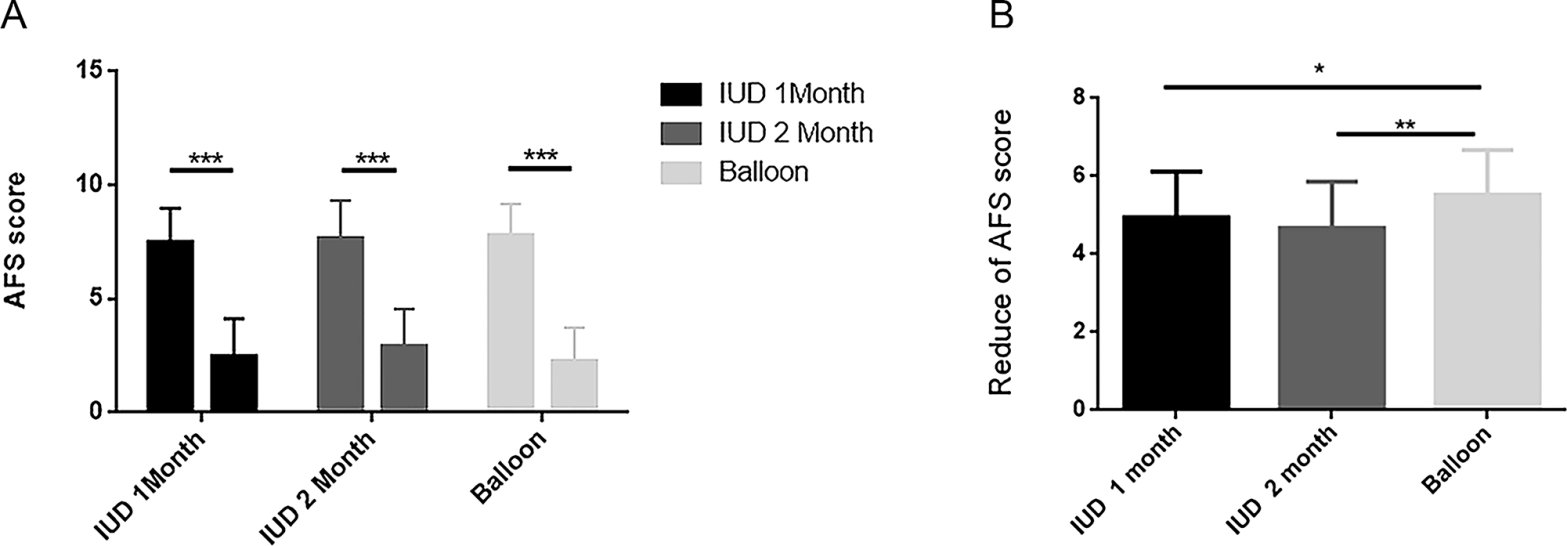
(**A**) Comparison of the mean adhesion score (MAFS) at first-look and second-look hysteroscopy between the three groups. (**B**) Comparison of the reduction in adhesion score between first-look and second-look hysteroscopy in the three groups

In the three groups, the AFS score at the second hysteroscopy was significantly lower compared with that prior to the initial operation (Fig. 2A). In addition, comparing the decreasing degree of the AFS score in each group exposed that the decline in the AFS score in the balloon group was greater than that in the IUD-1-month and IUD-2-month groups. Interestingly, no significant difference was observed between the two IUD groups (Fig. 2B).

### Endometrial thickness

**Fig. 3:**
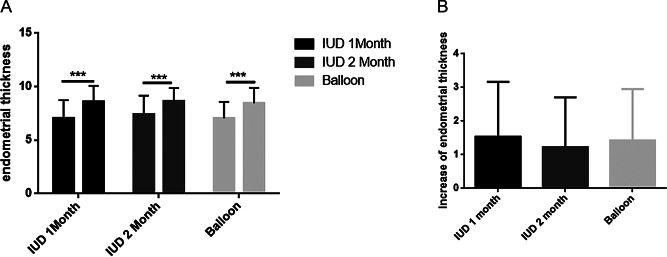
(**A**) Comparison of the mean endometrial thickness at first-look and second-look hysteroscopy between the three groups. (**B**) Comparison of the increase in endometrial thickness between first-look and second-look hysteroscopy across the three groups

Endometrial thickness was recorded during the late proliferative phase. Notably, the endometrial thickness in the three groups was significantly higher compared to that prior to the surgical operation (Fig. 3A). However, no significant difference was observed in the degree of increase in endometrial thickness among the three groups (Fig. 3B).

### Pregnancy outcomes

After the operation, patients underwent two D3 high-quality embryo transfers; 23 patients became pregnant in the balloon group, 25 patients became pregnant in the IUD-1-month group, and 24 patients became pregnant in the IUD-2-month group. Nevertheless, there was no significant difference in the pregnancy rate across the three groups. In addition, the rates of miscarriage, ectopic gestation, and premature delivery were similar among the three groups (see Table 3).

**Table 3:** Comparison of pregnancy outcomes among the three groups. There was no significant difference in pregnancy rate among the three groups. Likewise, the rate of miscarriage, ectopic gestation, and premature delivery were similar across the three groups

Group	IUD 1 month	IUD 2 month		Balloon	P value	X^2^
No Pregnancy	18 (41.8%)	19 (44.1%)	20 (46.5%)		0.189	0.910
Pregnancy	25 (58.2%)	24 (55.9%)	23 (53.5%)			
Miscarriage	3 (12.0%)	3 (12.5%)	4 (17.4%)		0.835	0.447
Ectopic Gestation	1 (4%)	3 (12.5%)	0 (0%)		0.210	2.998
Premature Delivery	4 (19.0%)	4 (22.2%)	4 (21.1%)		1.000	0.026
Live Birth	21 (48.8%)	18 (41.8%)	19 (44.2%)		0.803	0.439

Values are presented as numbers (%) unless otherwise indicated. X^2^ test; p-value of < 0.05 was considered statistically significant

## Discussion

IUAs frequently occur in the endometrium and myometrium after endometrial basal layer injury. The repair process includes three transient overlapping periods: the inflammatory phase, the tissue formation phase, and the tissue remodeling period. In severe IUA cases, the basal layer of the endometrium is disrupted [11], leading to a decline in endometrial regeneration capability and receptivity. Therefore, it exerts negative effects on women’s fertility, and patients with IUAs are generally associated with a low pregnancy rate or infertility. Currently, hysteroscopy, which can effectively separate adhesions under direct vision, has become the gold standard for IUA treatment [8]. Notwithstanding, the recurrence rate remains high, especially in cases of severe IUAs. Postoperative uterine readhesion is a key factor affecting not only postoperative outcomes but also the postoperative pregnancy rate. Thus, it is imperative to enhance postoperative uterine repair and prevent postoperative recurrence of IUAs. At present, several techniques have been developed to prevent readhesion after intrauterine adhesion separation, encompassing intrauterine devices (IUDs), balloons, hyaluronic acid polymers, and estrogen [8].

In 1966, Dr. Polishuk undertook the first study on the prevention of intrauterine adhesions using IUDS after TCRA. He postulated that the intrauterine placement of an IUD for 2–3 months can effectively prevent intrauterine adhesions [13]. At present, the majority of clinicians endorse the use of intrauterine devices to prevent intrauterine readhesion. Indeed, the implantation of IUDs into the uterine cavity has become the standard method for preserving the uterine cavity and is often used to prevent adhesion formation. Moreover, IUD promotes physiological endometrial regeneration by separating the anterior and posterior uterine walls [14]. Following TCRA, endometrial repair usually requires 1–2 months; consequently, IUDs are routinely placed for 2–3 months. However, no universal consensus has been reached on the optimal duration for IUD placement after TCRA. Herein, IUD placement for both 1 month and 2 months yielded satisfactory outcomes in preventing adhesion recurrence. Besides, the AFS scores were significantly lower at the second hysteroscopy, whereas the endometrial thickness was significantly higher. It is worthwhile emphasizing that the decline in the AFS score was not significantly different between the two IUD groups. Similarly, no significant difference was noted in the degree of increase in endometrial thickness between the two groups. The pregnancy rates in the two groups were also similar. An IUD is a foreign body in the uterine cavity that may cause excessive inflammatory reactions. Long-term placement may also cause abnormal bleeding, intrauterine infection, IUD incarceration, and uterine perforation [15, 16]. In our study, participants from three groups had similar severity of adhesions considering the AFS Scores appeared no significant difference. And afer analyzing raw datas, there became a result signalling that the duration of IUD placement may be shortened to reduce the risk of complications without compromising efficiency.

Given the evolving understanding of the mechanisms underlying intrauterine adhesions, clinicians may also consider placing a 3–5 mL balloon post-TCRA to prevent intrauterine readhesion. Placing a balloon as well as an IUD can act as a barrier for fresh wounds [17]. The balloon is larger in volume and facilitates endometrial proliferation along its surface. Additionally, postoperative uterine exudate can be directed outward along the urethra, and the balloon can suppress hemostasis [11]. An RCT study reported that a 7-day IUD and balloon placement after TCRA demonstrated similar efficacy in the prevention of adhesion recurrence [9]. Our study compared the efficacy of a 5-day balloon placement for one month and an IUD placement for two months. Our results revealed that the reduction in the AFS score in the balloon group was higher than that in the IUD-1-month and IUD-2-month groups, indicating that balloon therapy is superior in restoring uterine morphology and volume and minimizing the recurrence of IUAs. Nevertheless, the improvement in endometrial thickness and the pregnancy rate were comparable among the three groups.

Nevertheless, the relevant mechanism is unclear. The IUD acts as a physical barrier between the walls of the uterus, keeping them separated during the healing process. This reduces the likelihood of the surfaces sticking together and forming new adhesions. Additionally, IUDs are made of copper, and experimental evidence suggests that copper can improve inflammation by reducing the production of reactive oxygen species (ROS). This may also be one of the mechanisms by which IUDs ameliorate IUA [18]. 

To conclude, placing a balloon or placing an IUD for one month or two months can effectively prevent adhesion recurrence and restore the shape of the uterine cavity. However, balloon placement outperformed IUD in terms of therapeutic effect. Moreover, the three groups exhibited a significant increase in endometrial thickness following TCRA, but the increase in endometrial thickness did not substantially differ among the three groups. In addition, although these treatments may partially prevent IUAs, the pregnancy outcomes remain suboptimal. Thus, there is an urgent need to optimize postoperative uterine repair, prevent the postoperative recurrence of IUAs, and enhance the reproductive prognosis of patients with IUAs.

However, there still remained several limits. First and foremost, our study was conducted in a single tertiary university hospital and the sample size was small, which may limit this study. To further enhance the level of evidence in the study results, additional randomized controlled studies are warranted. Beyond that, in terms of the procedures conducted in our study, we failed to measure parcitipants’ compliance and standardization to hormone therapy and report complications and adverse events in detail, which played a crucial part in analyzing the comprehensive benefits and risks from receiving IUD or balloon therapy. And at the end of the study, there is a lack of long-term follow up about development of offsprings and qualitative feedback from patients, accounting for important considerations in clinical decision-making. Finally, in the subsequent experiments, we will add a group undergoing standard treatment to provide a clearer understanding of the efficacy of this therapy.

## Data Availability

The data that support the findings of this study are available from the corresponding author, Qing Zhang, upon reasonable request. Besides, research data has been upload into Mendeley Data. URL of the data repository site: https://data.mendeley.com/datasets/z7pvnmm5dh/1.
